# On the use of Bayesian decision theory for issuing natural hazard warnings

**DOI:** 10.1098/rspa.2016.0295

**Published:** 2016-10

**Authors:** T. Economou, D. B. Stephenson, J. C. Rougier, R. A. Neal, K. R. Mylne

**Affiliations:** 1 Department of Mathematics, University of Exeter, New North Road, Exeter EX4 4QE, UK; 2 Department of Mathematics, University of Bristol, University Walk, Bristol BS8 1TW, UK; 3 Met Office, FitzRoy Road, Exeter EX1 3PB, UK

**Keywords:** natural hazards, early warning system, decision theory, ensemble forecasting, ensemble post-processing

## Abstract

Warnings for natural hazards improve societal resilience and are a good example of decision-making under uncertainty. A warning system is only useful if well defined and thus understood by stakeholders. However, most operational warning systems are heuristic: not formally or transparently defined. Bayesian decision theory provides a framework for issuing warnings under uncertainty but has not been fully exploited. Here, a decision theoretic framework is proposed for hazard warnings. The framework allows any number of warning levels and future states of nature, and a mathematical model for constructing the necessary loss functions for both generic and specific end-users is described. The approach is illustrated using one-day ahead warnings of daily severe precipitation over the UK, and compared to the current decision tool used by the UK Met Office. A probability model is proposed to predict precipitation, given ensemble forecast information, and loss functions are constructed for two generic stakeholders: an end-user and a forecaster. Results show that the Met Office tool issues fewer high-level warnings compared with our system for the generic end-user, suggesting the former may not be suitable for risk averse end-users. In addition, raw ensemble forecasts are shown to be unreliable and result in higher losses from warnings.

## 1. Introduction

Early warning systems (EWSs) play a major role in reducing monetary, structural and human loss from natural hazards. The challenge of optimally issuing warnings is complicated—it is a ‘wicked’ problem [1] because the stakes are different for the entity responsible for issuing the warnings and the user receiving them. It is therefore beneficial to have shared ownership of the problem, facilitated by transparency of the EWS. A transparent and coherent framework for EWSs is required to encourage the engagement of all the involved stakeholders.

An EWS is defined here as a tool that uses (i) predictive information of the hazard and (ii) consequence (loss) information for each warning–outcome combination, to produce a warning according to some well-defined optimality criterion. It is a rule that transparently maps predictive and loss information into action. An EWS that is not transparently derived from well-defined inputs is defined here as ‘heuristic’.

Many operational EWSs, such as the Met Office National Severe Weather Warning Service (NSWWS) [2,3] and the flood warning system of the UK Environment Agency [4], are heuristic. The response to (and thus the overall effectiveness of) a warning system depends heavily on users believing that the warning is credible and accurate [5]. This belief is of course influenced by how well the system is formulated and understood. Agents that issue warnings suffer from the ‘cry-wolf’ syndrome, i.e. fear of loss of belief in the warning system due to false alarms; however, it has been argued that this is not necessarily true if the basis of the false alarm is well understood [6]. In other words, there are strong arguments for why an EWS should be as clear and transparent as possible. Such a system will also be amenable to criticism and thus improvement.

This article proposes a framework for issuing hazard warnings based on Bayesian decision theory [7], which offers a strategy for optimally issuing warnings in a rational way, using probability to quantify uncertainty about the future state of nature (hazard). We suggest a simple way of constructing the necessary loss functions for both generic and specific end-users, which provides a way of interpreting the warnings from the viewpoint of the decision-maker. We generalize previously proposed methodology to include any number of discrete warnings and future states of nature. The framework is illustrated by application to data from the UK Met Office first-guess warning system (a key component of the NSWWS) that uses predictive information in the form of ensemble forecasts (multiple predictions of potential future weather from a numerical weather model). We show how reliable probabilities for the future state of nature may be constructed from ensemble predictions and illustrate how the proposed EWS can also be used to quantify the value of various probabilistic predictions, for different stakeholders.

Section 2 defines the problem and briefly reviews relevant recent literature on natural hazard warnings. The decision theoretic approach is described in §3 and then applied to data from the current Met Office first-guess warning system for severe precipitation in §4. Section 5 concludes with a brief summary and a discussion.

## 2. Background

Issuing warnings for events such as severe weather or volcanic eruptions is a prime example of having to make real-time decisions under uncertainty. The uncertainty primarily comes from the fact that the occurrence and intensity of the future hazard are unknown and need to be predicted using complex yet imperfect models (e.g. the one described here in §4c). EWSs therefore rely on predictive information such as numerical model forecasts and observed precursors such as earthquake magnitude for predicting tsunamis. We define the set of all possible predictive information as *Y* with *y* being a particular value from this set. We also define the set of values that the state of nature can take as the state space *X* and the set of all possible actions as the action space *A*. For the agent that issues the warning, referred to here as the forecaster, action is defined as the decision of which warning to issue. For the end-user, it is protective action taken upon receiving a warning. The uncertainty in the prediction of a future *x*∈*X* is quantified by the conditional probability *p*(*x* | *y*). Losses for action *a*∈*A* are quantified using a loss function *L*(*a*,*x*)=ℓ_*a*,*x*_ which represents the loss incurred when action *a* is taken and then state of nature *x* subsequently occurs.

In prediction, where the goal is often to provide a best estimate of the future value *x*, the action space and the state space are the same. Relatively simple loss functions *L*(*a*,*x*) can then be used, for instance, a 0/1 loss where ℓ=0 only if the prediction comes true. In that case, it can be shown (using the Bayes rule defined in §3) that the optimal action is to predict *x* with the highest *p*(*x* | *y*). In a warning problem, the loss function cannot be so trivial and will likely be different for different stakeholders, for instance, the forecaster and end-user (e.g. a householder). Importantly however, the action set in the warning problem can be a lot more useful to stakeholders than the state space, since in practice, the action space will be considerably smaller—for instance, a finite set of warning levels compared to an infinite set of severe wind gust values. A good warning system can therefore be seen as the means by which forecasters and end-users communicate and share information—something that is particularly difficult due to the inherent uncertainty in the forecast (see, for instance [8] for challenges in communicating weather forecast uncertainty.)

Much of the scientific literature in natural hazards addresses the prediction problem, with a plethora of rigorous techniques and models, while the warning problem has received little attention and even less so with respect to decision theory. Sorensen [5] and Bhattacharya *et al.* [9] highlighted this in recent reviews of natural hazard and geohazard EWSs, and indicate the need for systems that integrate hazard evaluation and warning dissemination. In a paper discussing uncertainty in weather and climate information, Hirschberg *et al.* [10] also highlight the need for warning systems that are capable of using probabilistic forecasts. In the rest of this section, we present a review of some operational EWSs for natural hazards that address the warning problem, along with articles that have used decision theoretic approaches for both warning and prediction.

### (a) Review of decision theoretic approaches to natural hazard warning and prediction

There are numerous natural hazard EWSs in operation across the globe, e.g. for severe weather (such as the UK Met Office NSWWS, [2]), water-related hazards [11], hurricanes [12], Pacific tsunamis [13], volcanoes [14] and other geohazards [9]. A joint European effort for early warning of severe weather is made by National Meteorological offices through the website Meteoalarm [15]. All of these systems can be termed heuristic by our definition, and so (i) it is difficult to assess their utility for different users and (ii) it is unclear whether the rule for issuing warnings is optimal with respect to any loss function.

EWSs generally issue various levels of warning when the predicted probability of occurrence or the predicted magnitude of the hazard exceeds a certain threshold (see [16] where an earthquake alarm is triggered if the probability of intense ground motion is high enough). The thresholds are often chosen empirically, e.g. based on localized past damages to infrastructure. However, Martina *et al.* [17] used Bayesian decision theory to optimally estimate rainfall thresholds for issuing flood warnings on particular river sections.

Simple loss functions have been used to assess the value of weather forecasts (e.g. [18–20]). User actions and associated losses conditional on weather forecasts were considered, and the expected losses are used to evaluate the forecasts—as opposed to evaluating them solely on forecast skill. This can be considered a first step towards using decision theory for issuing warnings, as actions have losses attached to them. The second (missing) step is the strategy for taking optimal action, discussed in the subsequent section.

In Medina-Cetina & Nadim [21], a Bayesian network is used to integrate empirical, theoretical and subjective information into a probabilistic joint measure for the hazard. Although not designed as a tool for optimally issuing warnings, the method considers the event of issuing a warning given the available information as a stochastic node in the Bayesian network. This implies that the potential for a decision theoretic approach is there, if one were to extend the Bayesian network to an influence diagram by incorporating decision and utility nodes for the warnings [22].

Reynolds *et al.* [23] describes a decision support tool that uses probabilistic forecasts of cloud layer to minimize flight delays at the San Francisco airport. Different response scenarios were considered and the concept of a loss function was introduced, in order to select the scenario that minimizes expected loss.

Krzysztofowicz [24] is unique in explicitly advocating Bayesian decision theory as a way of issuing flood warnings. A flood forecasting system was proposed to estimate the probability of flood occurrence, which was then used in conjunction with a binary utility function of warnings to construct a rule that issues warnings to maximize expected utility. Here, we offer a more general framework to accommodate any number of warnings and states of nature, as well as a way of constructing the loss functions for the various stakeholders. As will be argued in §4, the loss function is the most crucial part of a Bayesian EWS, especially in terms of interpreting and assessing the warning rule. We also show how the conditional probabilities *p*(*x* | *y*) may be constructed from ensemble predictions.

## 3. A Bayesian approach to hazard warning systems

### (a) A framework for hazard warnings

Bayesian decision theory provides a coherent and transparent framework for making optimal warnings, using *p*(*x* | *y*) to express uncertainty about the future given predictive information *y*, and the loss function *L*(*a*,*x*) to quantify the consequences of the various actions *a*∈*A*. The theory provides an optimal decision rule a*(*y*) [25], a rule that maps *y* onto *A*, namely the Bayes rule, defined as

3.1 $$a^{*}(y)=\underset{a}{\text{arg min}}E[L(a,X)\;|\;Y=y]=\underset{a}{\text{arg min}}\int_{x}L(a,x)p(x\;|\;y)\;dx,$$

where E[⋅] denotes the expectation. In words, the optimal action a*(*y*), for given predictive information *y*, is to take action *a* that minimizes mean loss [26], ch. 11. So for a given set of actions *A* (e.g. levels of warning), the optimal action is a well-defined function of just two things: the loss function *L*(*a*,*x*) and the conditional probability *p*(*x* | *y*). If *x* is discrete, the integral in (3.1) is replaced by a sum.

**Figure 1.**
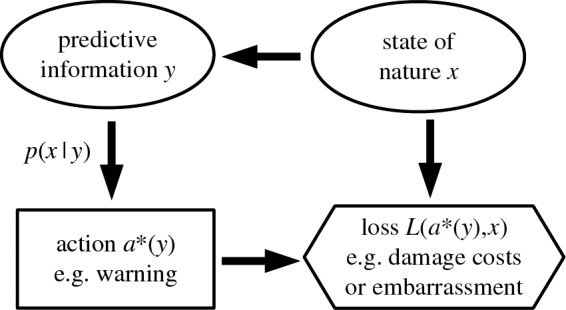
Influence diagram describing the decision problem of issuing hazard warnings. Oval nodes indicates uncertain quantities, rectangular nodes relate to decisions and hexagonal nodes relate to losses.

The Bayesian warning system can be depicted by an influence diagram [27] depicted in figure 1. The arrow from *x* to predictive information *y* captures the belief that predictions are actually related to the state of nature. The state of nature is not connected to the action node as it is unknown at the time that action is taken; only *y* is known and hence connected to the optimal action a*(*y*) through *p*(*x* | *y*). The loss function evaluating the consequence of issuing a warning is a function of a*(*y*) and the subsequent state of nature *x*.

To put things in context, consider the application in this paper which is the UK Met Office first-guess warning system introduced in §1, where *y* is an ensemble of *m* weather forecasts. The action space is a set of four increasing warning levels *A*={green,yellow,amber,red} and the state space is a set of severity categories of weather variables *X*={1,2,3,4}, the numbers corresponding to categories of an observable meteorological variable {very low, low, medium, high}, respectively. To formulate this problem using the proposed framework, the probability *p*(*x* | *y*) of the weather categories given the ensemble forecasts would first need to be estimated. This can be done using statistical modelling of historical pairs of observations of *x* and *y*, as described in §4c. Second, there is the non-trivial task of constructing the loss function, *L*(*a*,*x*), which here would be a 4×4 table shown in table 1. The values ℓ_*a*,*x*_ quantify the losses from issuing warning *a* (the letters *G*,*Y*,*A*,*R* being an alias for the four warning colours) when weather state *x* occurs, and will be different for different users of the system, e.g. the forecaster (issuer of the warning) and an end-user. Eliciting *L*(*a*,*x*) is the most difficult part of the assessment but equally the most important one: an agency responsible for issuing warnings is on shaky ground if it is not able to quantify losses and submit those losses to external scrutiny [22], ch. 1. Section 4 illustrates how the values in table 1 can be determined for generic stakeholders.

**Table 1.** Loss table *L*(*a*,*x*) for Met Office severe weather warnings.

		weather intensity *x*			
		1	2	3	4
warnings *a*	green	ℓ_*G*,1_	ℓ_*G*,2_	ℓ_*G*,3_	ℓ_*G*,4_
	yellow	ℓ_*Y*,1_	ℓ_*Y*,2_	ℓ_*Y*,3_	ℓ_*Y*,4_
	amber	ℓ_*A*,1_	ℓ_*A*,2_	ℓ_*A*,3_	ℓ_*A*,4_
	red	ℓ_*R*,1_	ℓ_*R*,2_	ℓ_*R*,3_	ℓ_*R*,4_

We can now ask if any heuristic decision rule is the Bayes rule for a particular loss function. If it is, then that loss function can be scrutinized and compared to other alternatives, for example, the loss functions proposed here in §4. If not, as is the case with the warning rule used by the UK Met Office described in the next section, then what is the justification for the decision rule if not decision theory?

Note also that a good decision rule can reduce loss and that will depend on how much the losses vary across actions in each state of nature, and also by how much this varies from state to state. In other words, the more sensitive losses are to the state of nature, the more useful a decision rule becomes. Having a large action space is a good way to increase the benefit from a well-designed decision rule such as the Bayes rule. Of course, the extent to which losses are reduced also depends on how well *y* predicts *x*.

## 4. Example: severe weather warnings

This section illustrates the Bayesian framework for issuing hazard warnings by application to precipitation data that was used in the first-guess NSWWS of the UK Met Office.

### (a) UK Met Office severe weather warning system

**Figure 2.**
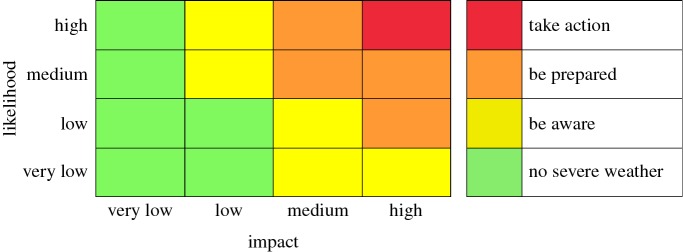
Likelihood-impact matrix that defines the Met Office warning rule used to construct warnings out of ensemble forecast information. ‘Impact’ refers to the magnitude of the forecasts and ‘likelihood’ refers to the relative frequency of this occurring in the ensemble forecasts. The likelihood categories are less than 20% for ‘very low’, 20–40% for ‘low’, 40–60% for ‘medium’ and more than 60% for ‘high’.

The UK Met Office NSWWS [2] provides warnings to civil responder services and the public using a risk-based ‘traffic light’ colour scheme where risk is assessed as a combination of likelihood and impact severity using the matrix illustrated in figure 2. The four warning levels (green, yellow, amber, red) are associated with top-level responder advice of ‘no severe weather’, ‘be aware’, ‘be prepared’ and ‘take action’. Warnings are issued subjectively by forecasters using a range of tools to assess the combination of likelihood and impact. Ensemble forecasting systems provide guidance on likelihood, but forecasters also make use of output from a range of forecast models. A numerical weather model is run many times with slightly different initial conditions to form an ensemble of predictions as a way of quantifying the uncertainty about the future state of weather (see [28] for some background on ensemble forecasting and [29] for probabilistic forecasting in general.) Impact is judged on a range of thresholds based on accumulated experience of aspects of societal vulnerability in different parts of the UK. Forecasters are also aided by an ensemble-based first-guess tool (used in this study) which uses the likelihood-impact table shown in figure 2, as the warning rule. The tool assesses the likelihood of severe weather impact categorized as ‘very low’, ‘low’, ‘medium’ and ‘high’ using a range of thresholds which vary geographically according to climate and vulnerability to represent impact. It assumes perfect forecasts so that the probability of say, a medium intensity event, is calculated as the empirical frequency of medium intensity from the ensemble members. The rule, which we shall refer to as MOrule, is then to choose the highest level warning from the table (see appendix Aa for a mathematical definition of the rule), e.g. if there is high likelihood of low impact weather (i.e. yellow warning) and a low likelihood of high impact weather (i.e. amber), then an amber warning is issued.

The MOrule is heuristic and not based on any explicit loss function (e.g. what is the consequence of a false alarm?) and hence it is not clear whether it is actually optimal in any way. Furthermore, the empirical forecast distribution *p*(*y*) is used instead of the conditional probability *p*(*x* | *y*) of the state of nature given the ensemble forecast information, i.e. numerical weather forecasts are assumed to be states of nature. In the rest of this section, we use historical data, to construct a Bayesian severe weather EWS as an alternative tool that does not suffer from those issues.

### (b) Data

The available data comprise 12-hourly observations of daily precipitation totals (in millimetre) for the county of Devon, along with matching forecasts, for the two extended winters of October 2012–March 2013 and October 2013–February 2014. The anticipated impact of precipitation is categorized as ‘very low’, ‘low’, ‘medium’ and ‘high’ (corresponding to *x*=1,2,3,4, respectively) for intervals 0–18, 18–25, 25–30 and >30 mm, respectively. Table 2 shows an example subset of the data. One-day ahead precipitation predictions are provided by the ensemble forecasting system of the European Centre for Medium-Range Weather Forecasts (ECMWF). This consists of an ensemble of *m*=51 forecasts of *x*(*t*) for any of the 12-hourly periods *t*. The forecast variable has eight categories defined by precipitation thresholds given in the bottom half of table 2 and is characterized by the vector *z*=(*z*_1_,*z*_2_,…,*z*_8_), where *z*_*k*_ is the number of ensemble members falling in category *k*. Note that information on individual ensemble members is not available—the data were provided in this categorical format, which was imposed in order to reduce storage space.

**Table 2.** Example of how observations (state of nature) *x* and the 8-category ensemble forecasts *z* are defined.

observations		very low 0–18		low 18–25	medium 25–30		high >30 mm	*x*
27/Oct/13 12:00UTC		0		1	0		0	2
28/Oct/13 00:00UTC		0		0	0		1	4
28/Oct/13 12:00UTC		1		0	0		0	1
⋯					⋯			

forecasts *z* thresholds	*z*_1_ 0–5	*z*_2_ 5–10	*z*_3_ 10–15	*z*_4_ 15–18	*z*_5_ 18–20	*z*_6_ 20–25	*z*_7_ 25–30	*z*_8_ >30 mm
27/Oct/13 12UTC	5	20	16	4	3	3	0	0
28/Oct/13 00UTC	0	0	0	0	2	3	11	35
28/Oct/13 12UTC	51	0	0	0	0	0	0	0
⋯					⋯			

The probability models described in the following section are estimated using 324 12-hourly values from the 2012–2013 extended winter period (the ‘estimation period’). The models are then used to sequentially predict *p*(*x* | *y*) and thus issue warnings for each of the 278 12-hourly values in the 2013–2014 winter (the ‘evaluation period’), updating the estimates accordingly after each 12-hourly prediction.

### (c) Simple probability models for *p*(*x* | *y*)

#### (i) Model CLIM

We start by quantifying the marginal probability *p*(*x*) as the empirical frequency of each of the four states of nature:

4.1 $$p(x=j)=\frac{n_{j}}{n},\;j=1,2,3,4,$$

where *n*_*j*_ is the number of observed *x* in category *j* out of *n* observations. For the estimation period, *p*(*x*)=(0.88,0.05,0.02,0.05). We denote this model as ‘CLIM’, as in the ‘climatological’ long-term frequency of *x*. Note that it is possible to use a longer historical record to estimate *p*(*x*) if appropriate, and one is not confined to using data that match the forecast values.

#### (ii) Model CAL

Before proceeding to consider the form of the predictive information *y*, we note that the forecasts *z* contain many zero values, e.g. *z*=(5,20,16,4,3,3,0,0) (first row of table 2) with corresponding relative frequency (0.1,0.39,0.31,0.08,0.06,0.06,0,0). Interpreting frequency as the forecast probability in each of the eight categories, implies that categories 7 and 8 are impossible. This does not reflect our belief that any category is possible at any time and we therefore apply ‘add-one smoothing’ (see [30], p. 79). The forecasts are therefore redefined as *z*′=(*z*_1_+1,…,*z*_8_+1). In the example of the first row of table 2, the new frequency is *z*′/(*m*+8)=(0.10,0.36,0.29,0.08,0.07,0.07,0.02,0.02).

For the sake of simplicity, we consider a simple univariate value as the predictive information *y*, that is representative of (forecast) precipitation intensity. We define *y*∈{1,…,8} as the modal label of *z*′. In other words, *y* is such that *z*′_*y*_≥*z*′_*k*_ for *k*=1,…,8, and in case of tied values, *y* is chosen as the label closest to the second-most-represented label.

We can now approximate the probability *p*(*y* | *x*) as the empirical frequency of *y* in each of the four *x* categories,

4.2 $$p(y=k\;|\;x=j)=\frac{n_{k,j}+1}{\sum_{k=1}^{8}(n_{k,j}+1)},$$

where *n*_*k*,*j*_ is the number of observed *y* taking the value *k* when observed *x* is in category *j*. Table 3 shows *n*_*k*,*j*_ for the estimation period showing that most of the data are concentrated at low values of *j* and *k*. Again add-one smoothing is used to reflect our belief that there is non-zero probability of a particular forecast category being dominant.

**Table 3.** Contingency table showing *n*_*k*,*j*_ for the estimation period. The columns correspond to counts in each of the four precipitation categories (*x*) and the rows correspond to counts in each of the eight forecast categories (*y*).

	observed precip. category			
	*j*=1	2	3	4
forecast precip. category				
*k*=1	209	2	0	0
2	53	6	1	1
3	18	8	4	4
4	3	0	1	1
5	0	0	0	0
6	1	1	0	6
7	0	0	0	4
8	0	0	0	1

Using Bayes’ theorem, we now have what is needed to calculate *p*(*x* | *y*), i.e.

4.3 $$p(x=j\;|\;y=k)=\frac{p(y=k\;|\;x=j)p(x=j)}{p(y=k)}=\frac{p(y=k\;|\;x=j)p(x=j)}{\sum_{j=1}^{4}p(y=k|x=j)p(x=j)},$$

which can be easily computed. We use ‘CAL’ to name this model, as in a model that ‘calibrates’ the forecasts, borrowing from the nomenclature in ensemble forecasting.

**Figure 3.**
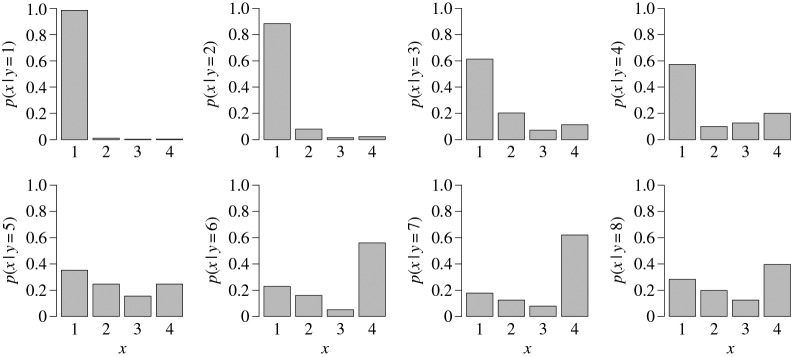
Estimates of the probability *p*(*x* | *y*) for each category of *y*=1,…,8, obtained using the simple calibration model CAL.

Figure 3 shows *p*(*x* | *y*) for each of the eight values of *y*, based on data from the estimation period. Note that add-one smoothing ensures that *p*(*x* | *y*) is well defined for each of the eight *y*-values. The plots suggest that there is more confidence in predicting *x* for low values of *y*, reflecting also the fact that the majority of the data are concentrated at low values of *x* and *y*. Overall, the probability of high precipitation categories seems to increase as the forecast categories increase.

#### (iii) ENS model

We also consider the model used by the Met Office first-guess tool, which assumes the ensemble forecasting system is a perfect representation of the state of nature. The four probabilities are estimated from the forecasts *z* as follows:

4.4 $$p(x=1\;|\;z)=\sum_{k=1}^{4}\frac{z_{k}}{m},\;p(x=2\;|\;z)=\sum_{k=5}^{6}\frac{z_{k}}{m},\;p(x=3\;|\;z)=\frac{z_{7}}{m}\;\mathrm{and}\;p(x=4\;|\;z)=\frac{z_{8}}{m}.$$

We call this model ‘ENS’, as it uses raw ‘ensemble’ frequencies.

### (d) Probability forecast performance

**Figure 4.**
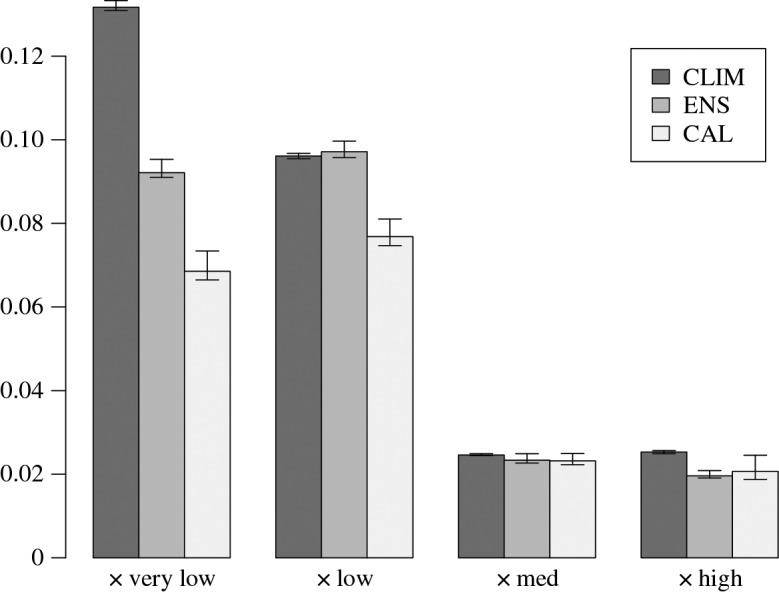
Barplot showing the Brier scores for each of the three probability models (CLIM, ENS and CAL) for each *x* category. The 95% bootstrap intervals, shown as ‘whiskers’ at the top of each bar, were calculated by re-sampling the data with replacement 5000 times.

The three models were used to sequentially predict precipitation in the evaluation period (2013–2014). After each prediction of a 12-hourly time step, models CLIM and CAL were updated accordingly, as would be done in an operational setting. The Brier score [31], a commonly used verification score for probability forecasts, was used to assess the predictive performance of each model:

4.5 $$B_{j}=\frac{1}{n}\sum_{t=1}^{n}{\left(\theta_{j}(t)-I(x(t)=j)\right)}^{2}\;j=1,\ldots ,4$$

where we use *θ*_*j*_(*t*) as the generic notation for the predicted probability of *x*(*t*)=*j* at time *t* given the forecast information, for instance, *θ*_*j*_(*t*)=*p*(*x*(*t*)=*j* | *y*(*t*)) for model CAL. Function I(x(t)=j) equals 1 if the observed *x*(*t*) equals *j*, and is zero otherwise. This is a ‘proper’ scoring rule widely used in forecast verification and smaller values imply higher forecast skill. The Brier scores for each precipitation category are shown in figure 4, indicating that model CAL has most skill, especially in the low categories. Approximate 95% confidence intervals for the scores, expressing estimation uncertainty, are illustrated as ‘whiskers’ (see appendix Ab for details). The intervals are smallest for CLIM and largest for CAL across all four categories illustrating the age-old trade-off between estimation uncertainty and model complexity.

**Figure 5.**
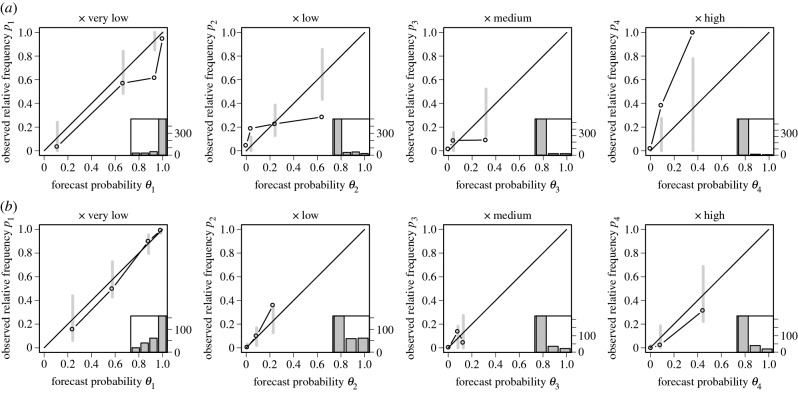
Reliability diagrams for model ENS (*a*) and model CAL (*b*). The histograms on the bottom right of each plot indicate the number of points in each bin used to construct the diagrams. The 95% consistency intervals indicate the variability in *p*_*j*_ that would be expected if forecasts were reliable.

We also assess the ‘reliability’ of the predicted probabilities. The probability forecast *θ*_*j*_, *j*=1,…,4 for the binary event *b*_*j*_=1 if *x*=*j* and *b*_*j*_=0 otherwise, is reliable if Pr(*b*_*j*_=1 | *θ*_*j*_)=*θ*_*j*_ [31]. In practice, however, even if the forecasting system is reliable, there will be discrepancies between *p*_*j*_=Pr(*b*_*j*_=1 | *θ*_*j*_) and *θ*_*j*_ since *p*_*j*_ has to be estimated from a limited amount of data. Reliability diagrams are plots of *p*_*j*_ against *θ*_*j*_ to visually assess how far points lie away from the *p*_*j*_=*θ*_*j*_ line (the diagonal). Figure 5 shows reliability diagrams for models ENS and CAL. The consistency bars that have been added along the diagonal (see appendix Ac for details) are such that for reliable forecasts the points should fall within the bars 95% of the time. The plots indicate that ENS is not an empirically reliable forecasting system (most points are outside the consistency bars), whereas CAL is. More specifically, ENS gives overly high probabilities for the high *x* category and too low probabilities for the less extreme categories.

### (e) A low-order parametric model of warning user loss functions

A loss function is essential for defining and constructing an optimum decision rule. It should faithfully represent a forecast user’s utilities for each of the possible combinations of state of nature and warning, e.g. 16 values for our example that has *J*=4 states of nature and *I*=4 warnings. Elicitation of so many values is not practical and so it is useful to have a simplified representation of the loss function that has only a few key parameters. We therefore propose here a simple parametric model for the loss function, which we believe captures the essential aspects for typical users of warning systems. While this parametric loss function can be used as is, it might also be used as the starting point for a more detailed assessment, where individual values are further adjusted. Sometimes it takes a ‘wrong’ value to flush out a better one.

To exploit properties such as monotonicity, it is useful to consider the elements of the loss matrix to be the discrete representation of a continuous function *L*(*a*,*x*) of *a*∈[0,1] and *x*∈[0,1], i.e. the loss in the *i*’th row and *j*’th column of the loss matrix is the loss *L*(*a*_*j*_,*x*_*i*_) defined at grid point *a*_*i*_=(*i*−1)/(*I*−1) and *x*_*j*_=(*j*−1)/(*J*−1) for *i*=1,2,…,*I* and *j*=1,2,…,*J*. This allows one to relate and compare loss matrices defined with different *I* and *J*.

The basic structure of *L*(*a*,*x*) can be identified by considering how a forecast user incurs losses. The two main reasons for losses are due to taking protective action once the warning is issued, and by having to pay for damages after an event occurs. The loss function can therefore be written as the sum of two parts: *L*(*a*,*x*)=*L*_*P*_(*a*,*x*)+*L*_*D*_(*a*,*x*). The protection loss, *L*_*P*_(*a*,*x*), occurs before *x* is known and so can only be a function of the warning *a*. Furthermore, it is reasonable to assume that protection loss increases with the magnitude of the warning, and so *L*_*P*_(*a*,*x*) is a monotonic increasing function *C*(*a*) of *a*. For simplicity, one can also assume that *L*_*D*_(*a*,*x*) is a separable function, i.e. *L*_*D*_(*a*,*x*)=*LR*(*a*)*D*(*x*), where *D*(*x*) is a monotonic increasing function of *x* (i.e. damage losses increase with the intensity of the experienced event) and *LR*(*a*) is a monotonic decreasing function of *a* (i.e. damage losses are reduced if a greater warning has been issued). Therefore, the basic form for the loss function is

4.6 L(a,x)=C(a)+LR(a)D(x),

where *C*(⋅) and *D*(⋅) are monotonic increasing functions and *LR*(⋅) is a monotonic decreasing function. Non-separable loss functions for damage can be constructed (if required) by adding additional terms to this low-rank tensor approximation of *L*(*a*,*x*).

To parametrize the loss function, it is necessary to specify functional forms for the three monotonic functions. One way to do this is to use power-law relationships such as

4.7 $$\begin{array}{rl} C(a) & =ca^{\gamma_{c}},\\ LR(a) & =l(1-a^{\gamma_{l}})\\ \;\text{and}\;D(x) & =x^{\gamma_{d}}, \end{array}\}$$

where *c* is the maximum prevention cost, *l* is the maximum damage loss and the shape parameters, *γ*_*c*_,*γ*_*l*_,*γ*_*d*_ are positive. The loss function is fully determined by the five parameters, *c*,*l*,*γ*_*c*_,*γ*_*l*_,*γ*_*d*_, which can be elicited for different users of the warning system. Appendix Ad presents analytic solutions for the Bayes rule and how it depends on the parameters for the continuum limit. In the special case where *I*=2 and *J*=2, this parametrization yields the simple binary cost-loss model described previously (e.g [19,24]) that has a decision rule which depends on the cost-loss ratio *c*/*l* and E(x | y).

Table 4 shows an example of a hypothetical loss function obtained with parameter values *c*=25, *l*=100, *γ*_*c*_=1.74, *γ*_*l*_=0.60, *γ*_*d*_=0.32 and its decomposition into protection and damage components. Such tables can easily be generated interactively for any chosen value of the parameters, which could then be used to elicit suitable parameter choices from specific users (e.g. via an online graphical interface). Such values could then subsequently be used by warning agencies to provide bespoke warnings that are optimal for each different user, e.g. by text message. Note also that in practice one can fix *l*, to say *l*=100, and then choose an appropriate cost loss ratio *c*/*l*, effectively reducing the number of parameters to 4. This is because *c* and *l* are arbitrary and it is the cost-loss ratio *c*/*l* that is important for determining the warning rule.

**Table 4.** Hypothetical loss function for the end-user. Top left panel shows protection loss *L*_*P*_(*a*,*x*), top right panel shows damage loss *L*_*D*_(*a*,*x*), and bottom left panel shows the overall loss function, i.e. *L*(*a*,*x*)=*L*_*P*_(*a*,*x*)+*L*_*D*_(*a*,*x*).

	protection loss *L*_*P*_(*a*,*x*)				damage loss *L*_*D*_(*a*,*x*)				overall loss *L*(*a*,*x*)			
	very low	low	medium	high	very low	low	medium	high	very low	low	medium	high
green	0	0	0	0	0	70	88	100	0	70	88	100
yellow	4	4	4	4	0	34	42	48	4	38	46	52
amber	12	12	12	12	0	15	19	22	12	28	31	34
red	25	25	25	25	0	0	0	0	25	25	25	25

To facilitate the elicitation of the loss function and to test sensitivity of the warning rule to the various inputs, we have provided an interactive tool written in the statistical software R [32] as electronic supplementary material. The four parameter values given above, were chosen (i) to reflect our beliefs about what the loss table for a generic end-user looks like and (ii) so that the resulting warning rule is robust to small changes in the four parameters. More generally, performing sensitivity analysis on the proposed loss function, we found that the resulting warning rule was most sensitive to the cost-loss ratio and whether or not *γ*_*c*_ and *γ*_*l*_ are close in value (see appendix A).

In addition to the forecast user, it is also of interest to imagine the reputational losses incurred by the forecaster for making false alarms and missed events. Table 5 shows a hypothetical example of what such embarrassment scores may look like for a forecaster. Note the zeros in the diagonal and the much higher loss for a red warning if *x* is very low, compared with the end-user—signifying that the end-user has more tolerance for false alarms. Unless user loss functions are clearly defined (and reported), it is possible that the forecaster may hedge warnings to be more optimal with respect to their own loss function. The parametrization of such loss functions and their decision-theoretic consequences could be a fruitful area of future research in forecast verification. An interesting point is how the interests of forecasters may be reconciled with those of end-users. As mentioned in §1, issuing warnings is a shared problem where both forecasters and end-users should have a say, and here we argue that the decision theoretic approach provides the necessary nexus through the language of loss functions.

**Table 5.** The loss function of a generic forecaster.

		weather intensity *x*			
		very low	low	medium	high
warnings	green	0	10	70	100
	yellow	20	0	10	70
	amber	50	10	0	10
	red	70	40	20	0

The loss functions in tables 4 and 5 do not necessarily reflect the losses for any particular individual, however, they do have to be visible thus allowing users to assess them, and even use them as a basis to construct their own loss function. The system can of course be adapted to any stakeholder that can provide their own loss function. In fact, it would be straightforward to develop an online service where the stakeholder inputs their own loss function, just once, and then receives bespoke warnings (e.g. by text message) based on *p*(*x* | *y*) provided say by the UK Met Office.

### (f) The warning rule

Using the loss functions in tables 4 and 5, and estimates of *p*(*x* | *y*) from model CAL based on the estimation period, the warning rules for the generic end-user and forecaster were computed and shown in table 6. The rules are quite different for the two stakeholders. No red warnings are ever issued by the forecaster, due to the combination of high losses from false alarms (bottom row of table 5) and high uncertainty in predicting *x* for high values of *y* as shown in figure 3. The end-user is more tolerant to false alarms and hence will receive higher warning levels than the forecaster across the range of *y*.

**Table 6.** Bayes’ warning rule for the generic end-user and forecaster.

modal label *y*	1	2	3	4	5	6	7	8
end-user	green	yellow	yellow	amber	amber	red	red	red
forecaster	green	green	green	yellow	amber	amber	amber	amber

**Figure 6.**
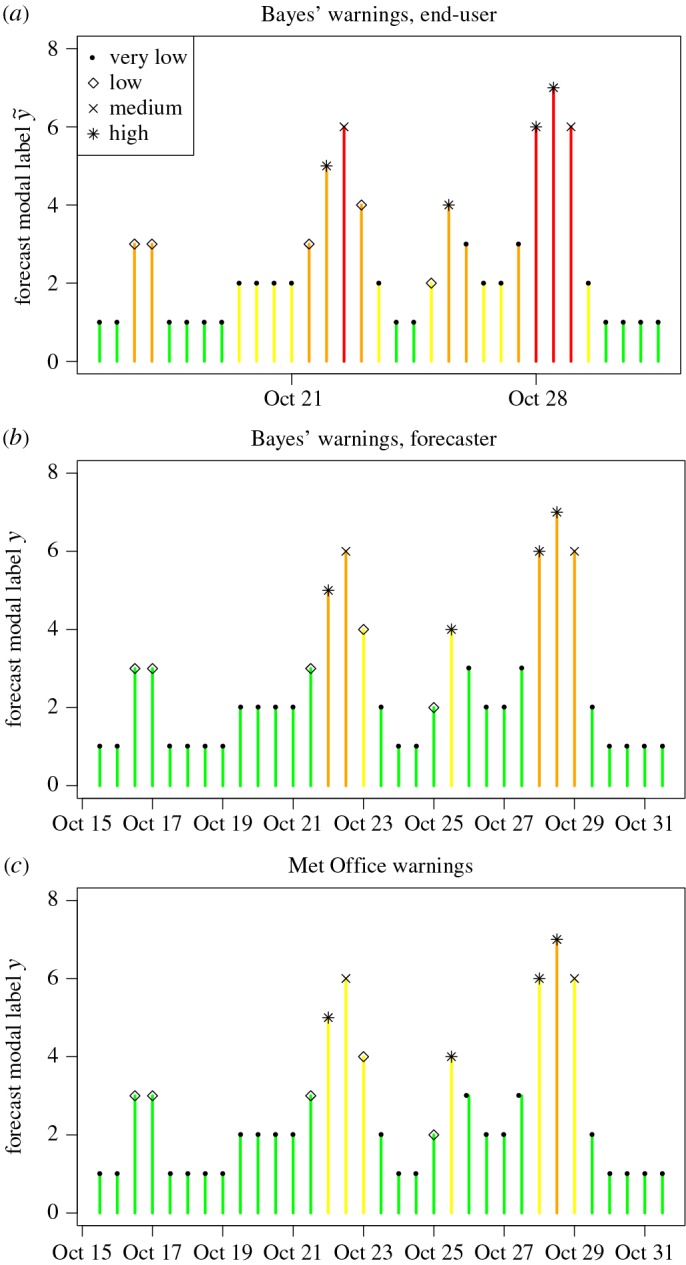
Plots of modal label *y* against time (12-hourly steps) for the last two weeks of October 2013 (the first month in the evaluation winter). Panels (*a*) and (*b*) show Bayes’ warnings for the end-user and forecaster, respectively. Panel (*c*) shows warnings based on the Met Office rule. The bars are coloured according to the warnings issued. The actual *x*-values are shown using symbols on top of each bar.

Figure 6 depicts Bayes’ warnings for the end-user and forecaster issued for the last two weeks of October 2013. The height of the bars indicates the value of *y* for each 12-hourly time step whereas the colour indicates the warning level. The symbols on top of each bar reflect the *x* category that actually occurred. Warnings issued using the MOrule are also shown in figure 6*c*. The plots indicate that the MOrule issues warnings similar to the generic forecaster who in turn issues fewer high levels of warnings than the generic end-user proposed here. In fact, the MOrule system only issued one red warning for the whole winter period (2013–2014).

**Figure 7.**
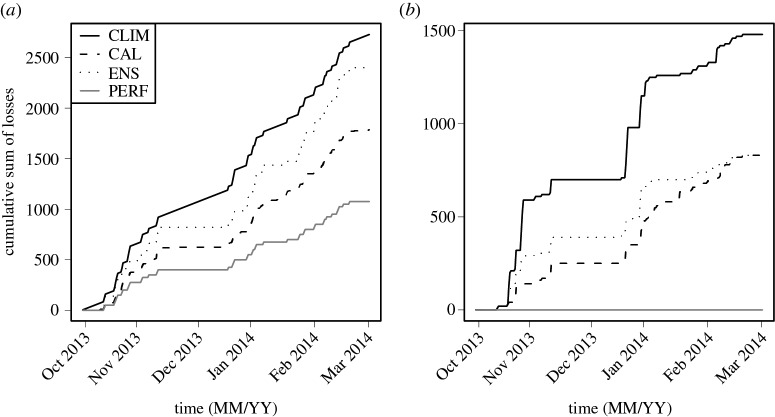
Plots of cumulative losses, for each generic stakeholder, that would have been incurred in the evaluation period (2013–2014), if warnings were issued using Bayes’ rule with probabilities from (i) model CLIM (solid line), (ii) model CAL (dashed line), (iii) model ENS (dotted line), and (iv) perfect knowledge (solid grey line). (*a*) End-user and (*b*) forecaster.

For each 12-hour time step in the evaluation period, figure 7 shows the end-user and forecaster accumulated losses that would have been incurred by issuing warnings from the Bayesian system with probabilities from (i) model CLIM (*p*(*x*)), (ii) model CAL (*p*(*x* | *y*)), (iii) model ENS (*p*(*x* | *z*)), and (iv) a model with perfect knowledge about the future (model PERF). Using climatological averages as probabilities resulted in the most losses, and while using raw ensemble forecast frequencies resulted in reducing those losses, it was model CAL that performed best. Note however that the difference in cumulative losses between models ENS and CAL is much less pronounced for the forecaster, indicating that using such generic loss functions can provide a way of comparing the value and potential usefulness of competing forecasting systems to various end-users (recall that model CAL only improved the Brier scores for the two lowest categories of *x* compared to model ENS). Using the interactive tool offered here as the electronic supplementary material, one can see that generally using CAL will result in smaller losses than ENS, which in turn results in smaller losses than CLIM, for most values of the four parameters defining the loss function for the end-user (§4e). The losses incurred by having perfect knowledge of the future provide a lowest loss bound on how much any system can improve by investing in better predicting *x*.

## 5. Discussion

Bayesian decision theory was proposed here as a transparent and natural framework for constructing and evaluating hazard warnings. The Bayesian EWS uses probabilistic predictions of the hazard in conjunction with a loss function to issue optimal warnings with respect to expected loss. Some methods for constructing and evaluating the probability of the hazard given relevant predictive information have been illustrated. In the application to precipitation warnings, the statistical model proposed to calibrate ensemble forecasts was shown to give smaller losses than simply using raw ensemble frequencies. It was also illustrated that quantifying consequences using a loss function is important in understanding and assessing the EWS.

The transparency of the proposed framework implies that it is open to criticism, updating and tailoring, which in turn means that it can accommodate likely changes in hazard forecasts, exposure and vulnerability. Expressing consequences numerically through a loss function, offers the interesting possibility of issuing bespoke warnings to different users with varying loss profiles.

Note that the framework proposed here can be incorporated into a decision support system in which a human agent makes the final decision. This decision will be based on Bayes rule, which the agent may choose to countermand on the basis of complexities that were not accounted for in predicting the probability of the future state of nature or in constructing the loss function.

The Bayesian model presented here to estimate *p*(*x* | *y*) was kept deliberately simple, in order to show that even with a simple model of *x* | *y* one can improve the accuracy of the predictions compared to using either *x* or *y* on their own. Sampling (parametric) uncertainty was not specifically accounted for, although techniques such as bootstrapping (appendix Ab) can be used to provide uncertainty intervals on the estimated probabilities. Here, the impact of this uncertainty on the decision rule was negligible, as indicated from sensitivity analyses performed using the provided interactive tool.

More complicated models can of course be developed, with the aim of improving the accuracy of *p*(*x* | *y*), bearing in mind, however, that increased model complexity can result in bigger estimation uncertainty as illustrated when looking at Brier score uncertainty in §4c. For instance, one can use conventional multinomial regression models as illustrated by Hemri *et al.* [33], who post-process categorical/ordinal variables. Potentially, the complete 8-category forecast variable *z* could be modelled in this way, instead of just the modal label. This should maximize the amount of information that can be obtained from the forecasts but it is left for future work. Ideally of course, information on individual ensemble members would be available, so that techniques such as kernel dressing or Bayesian model averaging could be used to obtain a smooth estimate of the ensemble distribution [34].

The Met Office first-guess warning system as presented here is a decision support tool. In practice, more than one ensemble forecasting system may be used as well as a deterministic system and the warnings actually issued are finalized by forecasters using subjective judgements and an assessment of societal vulnerability. The current warning level might have an effect on what warning will be issued next and forecasters will act upon their personal subjective beliefs and prior knowledge, adjusting the warning level as appropriate. Some of these particularities can be added to the proposed framework—for instance, considering information from other forecasting systems or even forecasts at different lead times as the predictive information *y* when building the model for *p*(*x* | *y*); or making the loss functions dynamically depend upon the current warning level. Not everything in the forecaster’s work can be replaced by a mathematical approach but at least the underlying system providing them with a suggested warning to issue should be transparent and defensible.

## Supplementary Material

Data and loss function tool

## Data accessibility

The Devon forecast and observation data used in this study as well as the R code to implement the interactive tool for eliciting parameters of the loss function are made available as the electronic supplementary material.

## Appendix A

**(a) Likelihood-impact matrix**

The Met Office rule (figure 2) is defined here mathematically. Suppose the weather variable of interest is *x* with support [*x*_*l*_,*x*_*u*_] and let the four *x* categories (very low, low, medium and high) be defined by intervals (*x*_*l*_,*x*_1_), (*x*1,*x*2), (*x*2,*x*3) and (*x*3,*x*_*u*_), respectively. The probabilities *θ*_*j*_=*p*(*x*=*j* | *z*) of falling in each interval *j*=1,…,4 given forecast information *z*, are obtained as the relative frequencies of ensemble members in each interval and are given by equation (4.4). Define also the relative frequency of exceedance above the three thresholds as: *f*_1_=1−*θ*_1_, *f*_2_=*θ*_3_+*θ*_4_ and *f*_3_=*θ*_4_. If we relabel the warnings {green,yellow,amber,red} into {1,2,3,4}, respectively, then the Met Office warning rule is

A 1 $$d(z)=1+I(f_{1}>0.4\;∥\;f_{2}>0\;∥\;f_{3}>0)+I(f_{2}>0.4\;∥\;f_{3}>0.2)+I(f_{3}>0.6),$$

where I(S) is 0/1 if *S* is true/false and the symbol ∥ denotes the logical statement ‘or’.

**(b) Brier score uncertainty**

For calculating Brier scores $B_{j}=(1/n)\sum_{t=1}^{n}{\left(\theta_{j}(t)-I(x(t)=j)\right)}^{2}$, the state of nature *x*(*t*) was assumed given so that only the uncertainty in estimating *θ*_*j*_(*t*) is assumed present. The uncertainty intervals of *B*_*j*_ were calculated by propagating the uncertainty in the *θ*_*j*_(*t*) estimates for each of the models CLIM, ENS and CAL.

**(i) Models CLIM and CAL**

The idea of bootstrapping was used to approximate confidence intervals for *B*_*j*_. The index *t*=1,…,*n* of observations *x*(*t*) and forecasts *y*(*t*), was sampled 1000 times with replacement, each time providing a new dataset (*x*^(*s*)^(*t*),*y*^(*s*)^(*t*)), *s*=1,…,1000. For each *s*, estimates $\theta_{j}^{\left(s\right)}=p(x=j)$ for CLIM and $\theta_{j}^{\left(s\right)}=p(x=j\;|\;y)$ for CAL were obtained and used to calculate $B_{j}^{\left(s\right)}$. The empirical quantiles of $B_{j}^{\left(s\right)}$ were used to approximate the 95% bootstrap intervals.

**(ii) Model ENS**

This model estimates *θ*(*t*) by (*e*_1_(*t*)+1,*e*_2_(*t*)+1,*e*_3_(*t*)+1,*e*_4_(*t*)+1)/(51+4), where *e*_*j*_(*t*) is the number of ensemble members in category *j* of the state of nature. Using add-one smoothing, is equivalent to a Bayesian approach assuming a flat Dirichlet prior for *θ*(*t*), *Dir*(*α*) with *α*=(1,1,1,1), so that the posterior is *Dir*(*α*′) with *α*′=(*e*_1_(*t*)+1,*e*_2_(*t*)+1,*e*_3_(*t*)+1,*e*_4_(*t*)+1). This posterior was sampled from 1000 times, each time calculating *B*_*j*_ to obtain a sample $B_{j}^{\left(s\right)}$. Empirical quantiles of this sample were used to construct the 95% credible intervals in figure 4.

**(c) Reliability diagram**

Consider forecast probabilities *θ*_*j*_(*t*), *t*=1,…,*n*, *j*=1,…,4, of binary events $b_{j}(t)=I(x(t)=j)$. A reliability diagram effectively plots *b*_*j*_(*t*) | *θ*_*j*_(*t*) against *θ*_*j*_(*t*). One way to achieve this is to bin *θ*_*j*_(*t*), then calculate ${\bar{b}}_{g}$ (the observed frequency of *b*_*j*_(*t*) in each bin *g*) and then plot ${\bar{b}}_{g}$ against ${\bar{\theta }}_{g}$ (the mean of *θ*_*j*_(*t*) in each *g*). If the forecasts are reliable, then the points on such a plot should lie ‘near’ the 45^°^ line, but not exactly due to sampling variability. 95% consistency bars can be added on the diagonal to assess how much the points would be expected to vary under the assumption of reliability. The R package ‘SpecsVerification’ [35] creates such bars by bootstrapping the forecasts *θ*_*j*_(*t*) and then simulating *z*_*j*_(*t*) under reliability (i.e. Pr(*z*_*j*_(*t*)=1)=*θ*_*j*_(*t*)). If too many points lie outside the consistency bars, then reliability can be rejected. See Broecker [31] and references therein for more details of reliability diagrams.

**(d) Optimal decision rules for the continuous loss function**

Insight into how the Bayes rule depends on the loss function parameters can be obtained analytically by considering the continuum limit of the loss function for an infinite number of states of nature and warnings, i.e. *a*∈[0,1] and *x*∈[0,1]. The optimal rule is given by substitution of equation (4.7) into equation (3.1)

A 2 $$a^{*}(y)=\underset{a}{\text{arg min}}(C(a)+LR(a)E[D(x)]),$$

where *C*(*a*) and *LR*(*a*) are monotonically increasing and decreasing functions, respectively. By differentiation, the minimum expected loss occurs when $C^{\prime }(a)+L^{\prime }(a)E[D(x)]=0$, where *C*′(*a*) and *A*′(*a*) are first derivatives wrt *a*. Substitution of the parametric forms in equation (4.7) then reveals that the minimum expected loss occurs at

A 3 $$a(y)={\left(\frac{\gamma_{L}}{\gamma_{C}}\frac{E[D(x)]}{c/l}\right)}^{1/(\gamma_{C}-\gamma_{L})}.$$

By considering the logarithm of both sides of this equation, it can be shown that the solution is in the interval *a*∈[0,1] only if either $\gamma_{C}>\gamma_{L}\max(1,\lambda )$ or $\gamma_{C}<\gamma_{L}\min(1,\lambda )$ where λ=E[D(x)]/(c/l). Hence, when *γ*_*C*_/*γ*_*L*_ is in the interval [min(1,λ),max(1,λ)], the minimum occurs at *a*∉[0,1] and so is not an acceptable warning. So, for example, when *γ*_*C*_=*γ*_*L*_, the minimum occurs at *a*∉[0,1] except in the highly unlikely case that *λ*=1 is satisfied exactly. When the local minimum occurs at *a*∉[0,1], the best warning rule then occurs at the boundary value of either *a*=0 if *λ*>1 or *a*=1 if *λ*<1, in other words, the optimal warnings are either to take no action whatsoever or take full action—any other intermediate warnings will lead to greater expected loss and so should not be issued. Sensitivity tests for discrete loss functions having *I*=4 and *J*=4 reveal similar difficulties in obtaining intermediate warnings when *γ*_*C*_ and *γ*_*L*_ do not differ substantially.
